# Synchronous Ovarian and Breast Cancers with a Novel Variant in BRCA2 Gene: A Case Report

**DOI:** 10.1155/2019/6958952

**Published:** 2019-01-06

**Authors:** Néstor Llinás-Quintero, Eduardo Cabrera-Florez, Gustavo Mendoza-Fandiño, Gustavo Matute-Turizo, Elsa M. Vasquez-Trespalacios, Luis J. Gallón-Villegas

**Affiliations:** ^1^Breast Surgery Fellowship Program, School of Medicine, CES University, Medellín, Colombia; ^2^School of Medicine, CES University, Medellín, Colombia; ^3^Departament of Public Health, School of Medicine, CES University, Medellín, Colombia; ^4^Pathology and Cytology Laboratory LLC (LAPACI), Medellín, Colombia; ^5^Epidemiology Department, School of Medicine, CES University, Medellín, Colombia

## Abstract

We report a case of a 52-year-old female with a family history of pancreatic and colon cancers who presented with a right breast mass positive for high-grade medullar carcinoma with triple-negative biomolecular profile. Further workup was performed finding a left ovarian mass. The patient underwent laparotomy performing optimal cytoreduction on bilateral ovarian tumors; the pathology and immunohistochemistry confirmed bilateral ovary adenocarcinoma with positive peritoneal malignancy. Due to her synchronic breast and ovarian cancers, a genetic profile was performed detecting a new pathogenic variant in the BRCA2 gene: c.3606_3607del (p.Ser1203Cysfs). She was given chemotherapy with carboplatin and paclitaxel obtaining complete clinical response. Regarding her breast cancer, she had a right modified radical mastectomy and prophylactic left mastectomy obtaining complete clinical response. This case presents with an unusual subtype and difficult histologic diagnosis of a synchronic medullar breast cancer and ovary carcinoma associated with a new mutation of the BRCA2 gene.

## 1. Case Report

**Figure 1:**
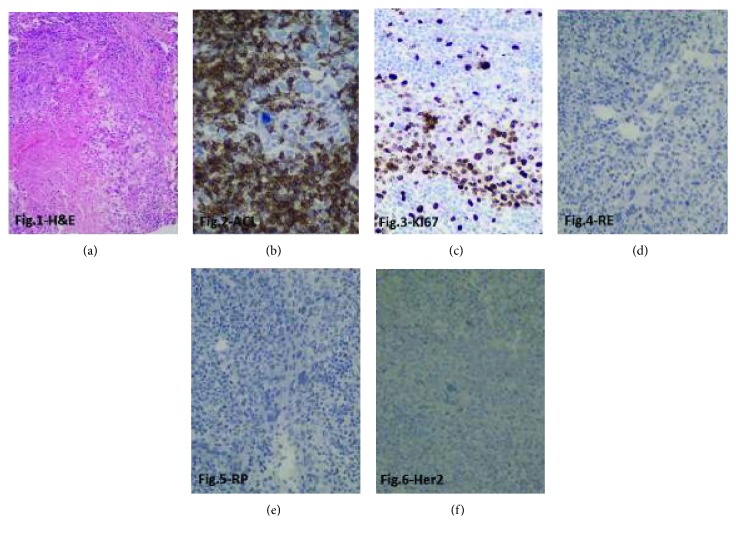
Immunohistochemical analysis of the breast lesion. (a) H&E 200x: central necrosis is observed, peripheral neoplastic cells with eosinophilic cytoplasm, large nuclei, prominent nucleoli, atypical mitosis, and mononuclear infiltrates. Representative micrographs (at 400x) are shown for immunohistochemical analysis of (b) leukocyte common antigen (LCA), showing positive expression in peritumoral lymphocytes. (c) Ki67 expression is detected in 60% of cancer cells; lack of expression of (d) estrogen receptor (ER), (e) progesterone receptor (PR), and (f) HER2.

A 52-year-old patient presented with a tumor on her right breast. Routine mammography classified it as BIRADS-0. A mammary ultrasound confirmed a complex cystic lesion in the upper quadrants of the right breast. Histopathological analysis of a biopsy of the tumor showed a poorly differentiated carcinoma with necrosis. An incisional biopsy of the breast tumor was performed and histopathology analysis reported it as a nuclear grade-3 medullary carcinoma. Pathology and immunohistochemistry of the lesion (60 × 40 mm) revealed an estrogen receptor negative (ER-), progesterone receptor negative (PR-), HER2 negative (HER2-), and Ki67 index of 20% mass (Figure 1), which led to the diagnosis of adenocarcinoma stage IIB (pT3N0M0) of the right breast.

We evaluated metastasis by chest X-rays and bone scintigraphy; no evidence of metastasis was identified. Abdominal ultrasound did not report liver lesions. A left ovary lesion was identified measuring 105 × 101 × 80 mm. The patient underwent exploratory laparotomy, which allowed the identification and resection of bilateral ovarian tumors. During the laparoscopic resection, analysis of the left ovary indicated an epithelial malignant tumor, consistent with breast cancer metastasis. Bilateral ovarian involvement by poorly differentiated adenocarcinoma was reported. Peritoneal cavity cytology identified additional malignancies, which by immunohistochemical studies were positive for the expression of WT1 and CA125 proteins suggesting an ovarian origin. The ovarian lesion was classified as serous cystadenocarcinoma. The patient underwent ovarian cancer staging surgery; uterine surgical pathology, remnant annexes, appendix, and omentum were negative for malignancy, obturator nodes were negative (0/11), and diaphragmatic cytology and parietocolic leakage were also negative. The final diagnosis was high-risk synchronous cancer, stage IIB triple-negative breast cancer, and stage IIIA ovarian cancer.

**Figure 2:**
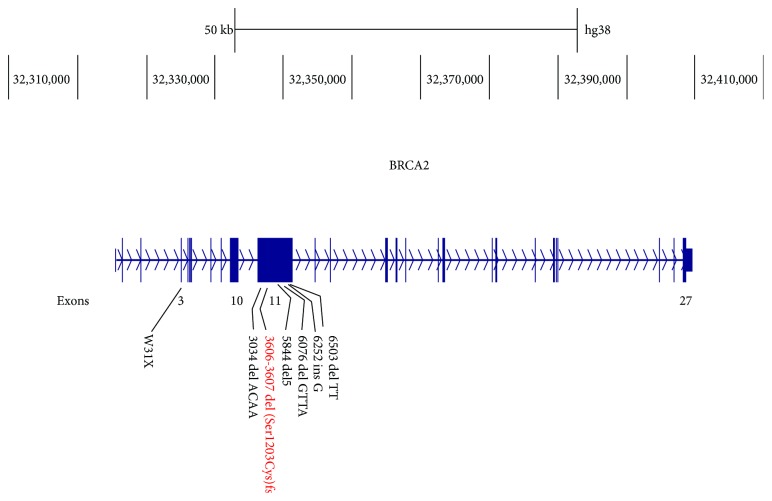
BRCA2 pathogenic variant: c.3606_3607del (p.Ser1203Cysfs).

The patient received adjuvant systemic therapy to treat the ovarian cancer and neoadjuvant therapy to treat the locally advanced breast cancer. The regimen was carboplatin AUC5+paclitaxel 175 mg/m^2^ day, every 21 days × 6 cycles, well tolerated, and exhibited complete clinical response. Genetic testing for BRCA1 or BRCA2 mutations (Myriad Genetics) reported a BRCA2 deleterious mutation: c.3606_3607del (p.Ser1203Cysfs) (Figure 2). The patient underwent bilateral mastectomy and axillary lymph node dissection. The final surgical pathology report indicated no residual disease, including 14 axillary ganglia, and a complete pathological response (ypT0N0M0). Coadjuvant radiotherapy was of 5000 cGy. Clinical follow-up after 33 months since diagnosis revealed no evidence of recurrent lesions and the patient reported her life quality as good.

Regarding the family history, a sister presents the same mutation in BRCA2: c.3606_3607del (p.Ser1203Cysfs). To our knowledge, their mother died with pancreatic cancer and the father was diagnosed with colon cancer. Family members are currently undergoing additional genetic tests.

## 2. Discussion

Triple-negative breast cancer (TNBC) and BRCA1/2-mutated breast cancers have been previously reported to exhibit sensitivity to platinum-based chemotherapy [1]. Thus, establishing BRACA1/2 status may be useful to provide a tailored chemotherapeutic regimen. The identification of patients at risk of being a carrier of BRCA1/2 mutation is relevant to hereditary cancer. BRCA1/2 mutation carriers are at high risk of breast cancer (RR > 10), similar to that observed in patients with a history of chest radiotherapy (usually lymphatic cancer, before 30 years old) [2, 3], breast surgery, systemic therapy, and other prophylactic interventions [4].

Furthermore, when designing the strategy and therapeutic approach for BRCA1/2 mutation carriers, the possibility of encountering synchronous and/or metachronous disease must be taken into consideration. However, when diagnosing synchronous disease, establishing whether it is a primary ovarian cancer or a breast cancer metastasis to the ovary may be challenging to determine. The additional possibility of it being a metastatic ovarian cancer to the breast is seldom found in the literature, and up to December 2015, only 110 cases have been reported [5, 6]. While criteria used to identify metastatic carcinomas—and differentiate them from primary tumors—are mainly based on clinicopathologic findings, loss of heterozygosity (LOH) and mutational analysis may provide useful additional information, since prognosis and therapy of those two entities are different [7]. Furthermore, breast cancer metastasis to the ovaries with a prevalence from 10 to 30% is associated with BRCA1/2 mutation carriers, which have worse prognosis, and is usually diagnosed during autopsy, prophylactic or therapeutic oophorectomies, and as incidental findings during routine surgery [8]. To date, metastatic breast cancer is generally identified histologically by the positive expression of gross cystic disease fluid protein 15 (GCDFP15), mammaglobin, and GATA3 and by the lack of expression of PAX8, CA125, and WT1. However, this is not always the case, since within the TNBC, the basal subtype exhibits low expression of GCDFP15 (11.9%) and mammaglobin (21.4%) [9]. Positive expression of CA125 and WT1 in metastatic breast cancer has also been reported [10]. Contrary to metastatic breast cancer, serous ovarian carcinoma shows positive expression of PAX8, CA125, and WT1 and lacks expression of (GCDFP15), mammaglobin, and GATA3. On the other hand, primary endometrioid ovarian cancer is usually identified by positive expression of CK7, estrogen receptor (ER), CA125, and PAX8, while lacking expression of CK20, CEA, and CDX2 [9–12].

Our patient was diagnosed with high-risk synchronous cancer: stage IIB TNBC and stage IIIA ovarian cancer. Immunohistochemical analysis in the ovarian tissue showed positive expression of CA125 and WT1. Because medullary carcinoma is uncommon, difficult to diagnose, and has significant interobserver variability, the National Comprehensive Care Network (NCCN) currently does not include a specific standard of care protocol for the treatment of medullary carcinoma. Nonetheless, the NCCN provides clinical guidelines for the treatment of medullary carcinomas, which are similar to those established for other infiltrating ductal carcinomas of comparable size, grade, and LN status. Additionally, medullary breast cancer has also been shown to have a comparable metastatic ability to that of other high-grade carcinomas. Therefore, our patient was treated with standard-of-care combination therapy for serous ovarian cancer (carboplatin+paclitaxel), as well as neoadjuvant therapy for BRCA2 mutation carrier TNBC patients [13, 14]. While there is currently no standard of care in the neoadjuvant setting for TNBC, and even to a lesser extent for BRCA1/2 mutation carrier breast cancer patients, several studies have reported the sensitivity of these tumors to platinum-based chemotherapy. The CALGB/Alliance 40603 and GeparSixto studies reported a pathological complete response (pCR) and an improved disease-free survival (DSF) for TNBC patients receiving carboplatin + standard of care in the neoadjuvant setting. Analysis of BRCA1/2 mutation carriers in those same studies showed no significant effect in pCR (52% and 4.7%, respectively), and while DFS was 85%, a greater toxicity was also reported for these patients [15, 16]. Preliminary results from a third phase II study reported that the addition of nab-paclitaxel to carboplatin led to a pCR of 53% [17]. Anthracycline-based therapy is considerably toxic, thus—aiming to reduce toxicity—other anthracycline-free regimens have also been studied. A study of 190 stage I-III TNBC revealed that patients (including BRCA1/2 germline mutation carriers) were treated in the neoadjuvant setting with carboplatin (AUC 6)+docetaxel (75 mg/m^2^), every 21 days, for 6 cycles. In that study, 16% of the patients were BRCA1/2 germline mutation carriers and exhibited a pCR of 59%, comparable to that observed by adding carboplatin to the anthracycline-taxane regimen [18]. Similarly, the phase II BSI-201 study (Telli et al.) showed that BRCA1/2 germline mutation carrier TNBC patients exhibited a pCR of 56% when treated with gemcitabine, carboplatin, and iniparib (GCI), comparable to that reported for TNBC patients receiving carboplatin+paclitaxel in the neoadjuvant setting [19–21]. The I-SPY2 study found a pCR of 52% in TNBC patients treated with carboplatin/veliparib in combination with paclitaxel and anthracycline-based chemotherapy [22]. Finally, additional studies have reported a pCR ranging from 90 to 100% using cisplatin as single neoadjuvant agent for BRACA1 mutation carrier TNBC patients [23–25]. While carboplatin+taxane therapy in the neoadjuvant setting remains to be fully demonstrated in order to be implemented as standard of care for TNBC patients, it may be a valid option to improve the pCR rates and DFS in those patients carrying BRCA mutations.

Thus, the search for BRCA1/2 pathogenic variants associated with breast cancer is of clinical significance for the individual patient, as well as for the patient's relatives. In this case report, we have identified the pathogenic BRCA2 variant (c.3606_3607del (p.Ser1203Cysfs)), which—to our knowledge—has not been previously reported. The genetic screening of her relatives is an ongoing study at our institution. Medullary breast carcinomas (MBC) share specific genomic characteristics. Transcriptomic profiles revealed that MBC differ from non-MBC with 92 genes overexpressed and 154 genes underexpressed in MBC [26]. Differences in molecular characteristics between MBC and invasive ductal tumors with a basal-like phenotype may account for the relative favorable outcome for MBC [27]. MBC have been reported more frequently in Afro-American patients. Liao et al., in their multivariate analysis, taking infiltrating ductal carcinoma as a reference, found that patients with medullary or apocrine carcinoma had excellent prognosis and that patients with metaplastic or mixed lobular-ductal carcinoma had poor survival outcomes [28]. With regard to clinical characteristics of MBC, some studies have reported a lower mean age in MBC patients and mixed ductal-lobular compared with other TNBC subtypes. Lymph node status does not show statistical differences according to the histological subtype, but this may be due to small sample sizes [29]; MBC had more limited stage and smaller tumors at presentation in a study assessing the histologic heterogeneity of triple-negative breast cancer [30]. Considering local invasion, MBC seem to have a less aggressive manner compared to invasive ductal carcinoma [31]. Overall survival of MBC patients was higher when compared to that of invasive ductal carcinoma patients [32, 33]. Zangouri et al. found a significant statistical difference between invasive ductal carcinoma and MBC (92.8% vs. 98.1%, *P* = 0.004) and also with the 5-year overall survival rate (86.3% vs. 94.2%, *P* = 0.008) [31]. According to these differences, a uniform approach may be not the best choice.
